# Fahr's Syndrome With Hypoparathyroidism in a Patient With Schizophrenia and Iron Deficiency Anemia: A Case Report

**DOI:** 10.1002/ccr3.70093

**Published:** 2025-01-06

**Authors:** Ali Haider, Ali Danish, Allahdad Khan, Atta Ur Rehman, Muhammad Haris Latif, Salman Abdul Basit, Aseel Kamal, Obaid Imtiyazul Haque

**Affiliations:** ^1^ Department of Medicine Nishtar Medical University Multan Pakistan; ^2^ Southwest Healthcare System, Wildomar California USA; ^3^ SSM Health Saint Louis University Hospital St Louis Missouri USA; ^4^ Department of Internal Medicine Wright Center for Graduate Medical Education Scranton Pennsylvania USA; ^5^ Faculty of Medicine University of Gezira Faculty of Medicine Wad Madani Gezira Sudan; ^6^ Department of Medicine MedStar Union Memorial Hospital Baltimore Maryland USA

**Keywords:** basal ganglia, bilateral calcification, Fahr's syndrome, hypocalcemia, hypoparathyroidism, schizophrenia

## Abstract

Fahr's syndrome is a rare neurological disorder that shows up as calcium deposits in the brain, affecting motor control and cognitive functions. In this case report, a 45‐year‐old woman with schizophrenia was diagnosed with Fahr's syndrome, which can be challenging to diagnose due to coexisting neurological comorbidity.

## Graphical abstract

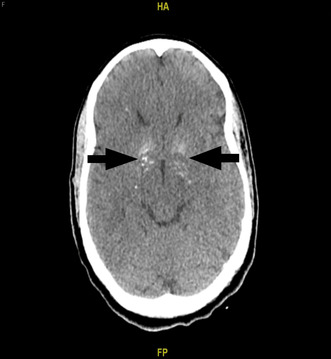

## 1 Introduction

Fahr's syndrome or primary familial brain calcification (PFBC) is a rare and progressive neurodegenerative disorder, which is characterized by calcification of the brain in many areas, particularly in individuals in their third or fourth decade of life [1]. Fahr's syndrome has a prevalence of less than 1 in 1,000,000 and is a diagnostic and clinical dilemma because of its rarity and varied presentations [2].

Hypoparathyroidism is a rare endocrine disease resulting from dysfunction of the parathyroid glands and deficiency of parathyroid hormone (PTH). Later, because of PTH deficiency or decreased levels of the hormone, hypocalcemia and hyperphosphatemia occur and are followed by neuromuscular irritability. It is very common for patients to present with hypocalcemia symptoms such as myalgias, muscle spasms, twitches, new‐onset seizures, and rarely tetany [3].

Hypoparathyroidism that is defined by low serum calcium and PTH deficiency is one of the causes of Fahr's syndrome. Chronic hypocalcemia leads to ectopic calcification in the brain worsening neurological and mental disturbances [4].

Fahr's syndrome is rarely observed to coexist with psychiatric conditions, though in some instances it may pose a diagnostic dilemma, as the neuropsychiatric alterations may mimic primary psychiatric disorders such as schizophrenia.

In this case, a patient with Fahr's syndrome, accompanied by hypoparathyroidism, schizophrenia, and iron deficiency anemia, is described. This case illustrates the need to consider secondary causes of intracranial calcifications in patients with psychiatric features, to avoid missing the underlying metabolic disorder presented as the psychiatric manifestations, and to support the need for a multimodal approach in managing patients with both metabolic and psychiatric comorbidity.

### 1.1 Case History / Examination

A 45‐year‐old female with a past medical history of iron deficiency anemia and schizophrenia came to the emergency department complaining of loss of consciousness while on a walk. The patient fell, sustaining a 1‐in. full‐thickness laceration to the occipital region. The patient also had an episode of tonic‐clonic seizure of unknown duration after the fall, according to the patient's history. At the time of the incident, the patient was not on any medication.

The patient's blood pressure was 139/80 mmHg using a digital sphygmomanometer, pulse rate 80/min, respiratory rate 17/min, and SpO_2_ 98% on room air. The patient denied any history of seizures, dizziness, or blurred vision before the incident. There were no signs of tongue bite, fecal, or urinary incontinence.

Neurological examination revealed that the patient was alert and fully conscious, with a Glasgow Coma Scale (GCS) score of 15/15. Somatosensory function was intact, with normal motor strength (5/5) and reflexes (2+). Coordination and gait were normal, indicating no neurological abnormalities.

## 2 Methods (Differential Diagnosis, Investigations and Treatment)

On investigation, the patient had hypocalcemia (5.0 mg/dL), and toxicology was positive for cannabinoids only. Electroencephalogram (EEG) was unremarkable. The cervical spine CT scan was negative for any abnormality, and the chest and pelvis X‐rays revealed no acute osseous abnormalities. Echocardiography showed an ejection fraction of 55%–60% with normal left ventricular systolic function. Electrocardiogram (ECG) showed diffuse T‐wave inversion with no acute events on telemetry.

On admission, her calcium was 5.0 mg/dL (Table 1). Her PTH was low at 9.4 pg/mL, and vitamin D 25‐OH was 17.7 ng/mL. Phosphorus levels were high at 6. The patient's hemoglobin was 7.2 g/dL upon admission. Fecal occult blood was negative.

**TABLE 1:** Laboratory test results of the patient.

Lab test	Result	Unit
Random blood sugar	131	mg/dL
Serum calcium	5.0	mg/dL
Serum ionized Ca	3.1	fL
Magnesium	1.8	mg/dL
Alanine aminotransferase	13	U/L
Aspartate aminotransferase	21	U/L
Alkaline phosphatase	53	U/L
Estimated glomerular filtration rate	76.3	mL/min/1.73 m^2^
Ferritin	9	ng/mL
Intact parathyroid hormone	9.4	pg/mL
Vitamin D 25‐OH	17.7	ng/mL
Iron	13	μg/dL
Total iron binding capacity	292	μg/dL
Transferrin saturation	4%	—
Vitamin B12	597	pg/mL
Hemoglobin	7.2	g/dL
Red blood cells	3.84	x10^6/μL
Hematocrit	25	%
Mean corpuscular volume	65	fL
Red cell distribution width	23.1	%
Neutrophils	38.7	%
Lymphocytes	9.8	%
Monocytes	1.1	%
Eosinophils	1.7	%
Basophils	1.1	%

The fall was ultimately attributed to an isolated seizure caused by severe hypocalcemia, rather than Fahr's syndrome or anemia.

The patient was admitted for further management of hypocalcemia and anemia. Intravenous calcium gluconate was administered, raising serum calcium levels to 7.0 mg/dL within 2 days. Iron deficiency anemia was addressed with intravenous iron (ferrous sulfate 400 mg/day), and two units of packed red blood cells were transfused, improving hemoglobin levels to 9.7 g/dL. The hemoglobin level was corrected to 9.7 g/dL and remained stable.

## 3 Conclusions and Results (Outcome and Follow‐Up)

**FIGURE 1:**
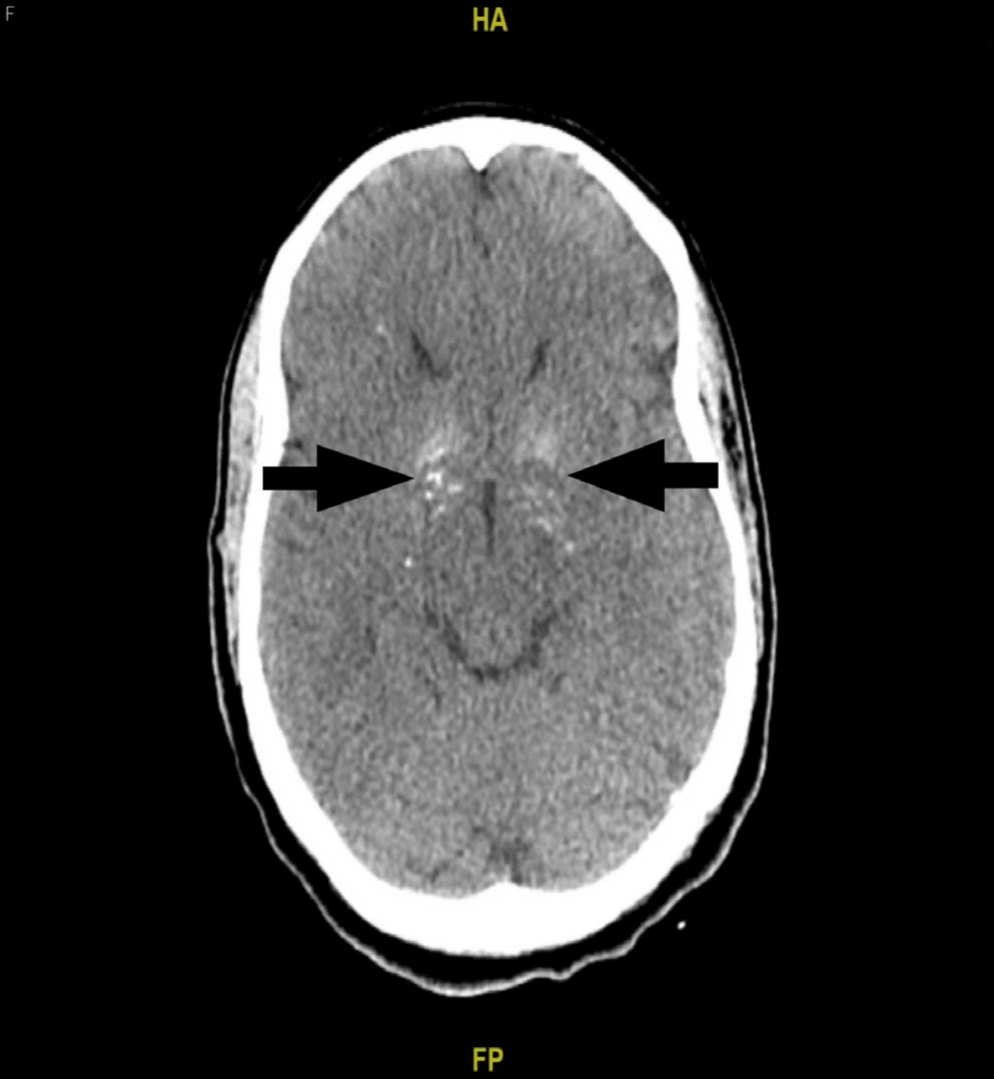
Brain CT scan showing bilateral basal ganglia calcification presenting as hyperdense areas in the basal ganglia region.

**FIGURE 2:**
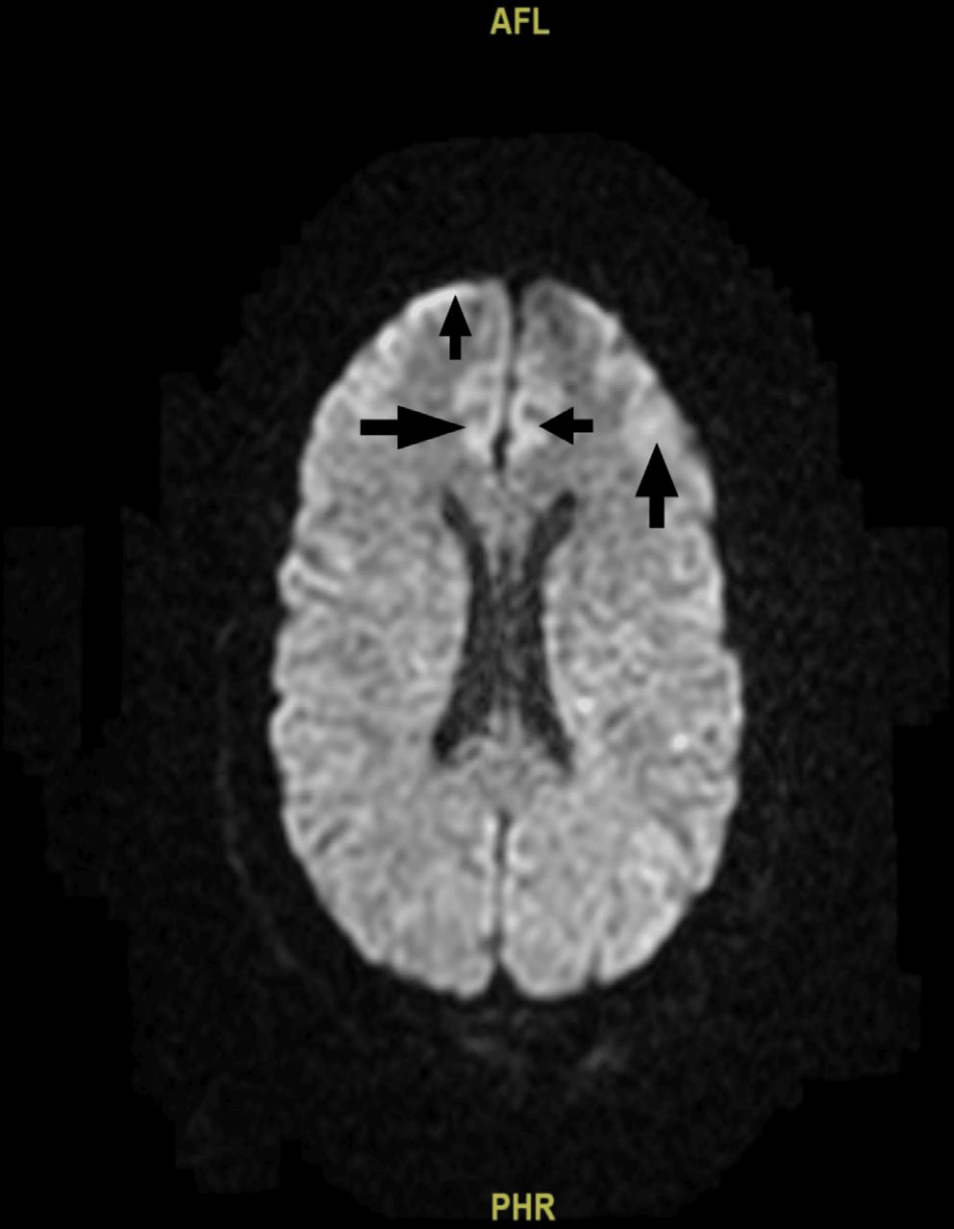
Brain MRI showing bilateral basal ganglia mineralization, mainly in the pallidum, with sparing of the caudate head, and small mineralizations in the frontal lobe white matter, centrum semiovale, and cerebellar hemispheres.

A brain CT scan (Figure 1) showed bilateral basal ganglia calcifications as hyperdense areas. Followed by a brain MRI (Figure 2) for further workup of hypoparathyroidism, it showed basal ganglia mineralization with relative sparing of the caudate head and predominantly involving the pallidum, with associated small areas of white matter mineralization in the deep white matter of the frontal lobes bilaterally in the centrum semiovale and the white matter of the cerebellar hemispheres bilaterally. A cardiology consultation recommended the use of an extended‐event monitor or loop recorder. Routine outpatient follow‐ups were advised, along with avoidance of medications that could prolong the QT interval. The patient was discharged with recommendations for follow‐up with an endocrinologist, a cardiologist, and her primary care physician to address her findings.

## 4 Discussion

Both Fahr's syndrome or PFBC and schizophrenia cause diagnostic dilemmas and require stringent management due to comparable clinical manifestations and the involvement of multiple factor pathways. Fahr's syndrome is associated with calcification in the cerebral tissue, more frequently in the basal ganglia, thalamus, and cerebellum [2]. This calcification is connected with genes that code for phosphate regulation and vascular tropism including SLC20A2, PDGFB, PDGFRB, and XPR1 [5, 6].

Psychiatric diseases such as schizophrenia, on the other hand, are classified as neurodevelopmental disorders with polygenic and polyetiologic origins that involve genetic vulnerability, neural transmitter imbalance, and many different environmental factors. This is because both Fahr's syndrome and psychiatric illness have a broad range of neurological/psychiatric manifestations making the diagnosis challenging. Fahr's syndrome symptoms range from movement disorders, learning disability, and epilepsy to various types of psychosis and depression [7]. In the same way, schizophrenia is associated with hallucinations, paranoid and bizarre delusions, poor cognitive functioning, as well as deficits in motivation, emotional expression, and social engagement.

Fahr's syndrome diagnosis usually involves imaging studies, computerized tomography, and magnetic resonance imaging to demonstrate the presence of the abovementioned calcifications together with metabolic and genetic tests to establish the etiology [8]. In contrast, psychiatric diseases have symptoms that are systematically described in manuals, such as the DSM‐5, with no specific markers to diagnose the diseases. Fahr's syndrome requires the following treatments because its management is mainly symptomatic: hypocalcemia requires calcium and vitamin D supplementation, seizures require appropriate medications, and psychiatric symptoms, which include psychosis, require antipsychotic medication. The management involves neurologists, psychiatrists, and endocrinologists, and their involvement is necessary for the best outcome. On the other hand, a disorder such as schizophrenia involves treatment with antipsychotic agents, psychosocial interventions, and supportive care, which are targeted to enhance functional recovery.

Fahr's syndrome has no cure and its outcome depends on the severity and progression of symptoms, with focused attention on early intervention to manage metabolic changes that could otherwise lead to neurological decline. Schizophrenia and other similar psychiatric disorders are typically characterized by a relapsing–remitting nature, and treatment management aims at minimizing symptoms and the level of disability in the long term. Comparing Fahr's syndrome with mental disorders is critical for clinicians to broaden their knowledge and make the right diagnoses and treatment plans when dealing with clients diagnosed with these conditions. Each needs an intensive, integrated, and holistic approach in terms of diagnostic approaches and therapeutic management to enhance patients' quality of life. It is critical to proceed with future research aimed at identifying the pathophysiological pathways of these multifactorial conditions and their effective management.

## Author Contributions

Conceptualization, data curation, investigation, methodology, resources, supervision, writing – original draft: Ali Haider. Data curation, investigation, methodology, project administration, validation, visualization, writing – original draft: Ali Danish. Conceptualization, formal analysis, methodology, visualization, writing – original draft, writing – review and editing: Allahdad Khan. Data curation, investigation, resources, software, writing – original draft: Atta Ur Rehman. Investigation, methodology, project administration, validation, visualization, writing – original draft: Muhammad Haris Latif. Data curation, formal analysis, investigation, resources, software, writing – original draft: Salman Abdul Basit. Conceptualization, data curation, methodology, project administration, writing – original draft, writing – review and editing: Aseel Kamal. Conceptualization, data curation, supervision, validation, visualization, writing – review an editing: Obaid Imtiyazul Haque.

## Consent

Written consent from the patient was obtained.

## Conflicts of Interest

The authors declare no conflicts of interest.

## Data Availability

Data available on request from the authors.
